# Nanoparticulate vacuolar ATPase blocker exhibits potent host-targeted antiviral activity against feline coronavirus

**DOI:** 10.1038/s41598-017-13316-0

**Published:** 2017-10-12

**Authors:** Che-Ming Jack Hu, Wei-Shan Chang, Zih-Syun Fang, You-Ting Chen, Wen-Lin Wang, Hsiao-Han Tsai, Ling-Ling Chueh, Tomomi Takano, Tsutomu Hohdatsu, Hui-Wen Chen

**Affiliations:** 1 Institute of Biomedical Sciences, Academia Sinica, Taipei, Taiwan; 2 Research Center for Nanotechnology and Infectious Diseases, Taipei, Taiwan; 3 Department of Veterinary Medicine, National Taiwan University, Taipei, Taiwan; 4 School of Veterinary Medicine, Kitasato University, Towada, Aomori, Japan

## Abstract

Feline infectious peritonitis (FIP), caused by a mutated feline coronavirus, is one of the most serious and fatal viral diseases in cats. The disease remains incurable, and there is no effective vaccine available. In light of the pathogenic mechanism of feline coronavirus that relies on endosomal acidification for cytoplasmic entry, a novel vacuolar ATPase blocker, diphyllin, and its nanoformulation are herein investigated for their antiviral activity against the type II feline infectious peritonitis virus (FIPV). Experimental results show that diphyllin dose-dependently inhibits endosomal acidification in fcwf-4 cells, alters the cellular susceptibility to FIPV, and inhibits the downstream virus replication. In addition, diphyllin delivered by polymeric nanoparticles consisting of poly(ethylene glycol)-block-poly(lactide-co-glycolide) (PEG-PLGA) further demonstrates an improved safety profile and enhanced inhibitory activity against FIPV. In an *in vitro* model of antibody-dependent enhancement of FIPV infection, diphyllin nanoparticles showed a prominent antiviral effect against the feline coronavirus. In addition, the diphyllin nanoparticles were well tolerated in mice following high-dose intravenous administration. This study highlights the therapeutic potential of diphyllin and its nanoformulation for the treatment of FIP.

## Introduction

Feline coronaviruses (FCoVs) belong to the genus *alphacoronavirus* in the family *Coronaviridae*. As a ubiquitous viral pathogen among domestic and wild feline populations, FCoV can be horizontally transmitted via the fecal-oral route with persistent infection, and the prevalence and seropositivity in multi-cat households or shelters may achieve 90%^1,2^. With regard to pathogenicity, FCoV exists as two biotypes, namely feline enteric coronavirus (FECV) and feline infectious peritonitis virus (FIPV)^3^. Genetic mutations occurred in FECV within a persistently infected cat allow it to acquire macrophage tropism and change the virus into the virulent FIPV^4^. Infections caused by the former show no clinically apparent or mild, self-limiting gastroenteritis in kittens. In contrast, FIPV infections develop high fatality and progressive, multi-systemic disorders in young cat populations. Feline infectious peritonitis remains one of the most devastating and lethal viral diseases among *Felidae*, not only because of the high mortality rate, but also because of the lack of effective options in diagnosis, prevention, and treatment^5^. The disease is further complicated by antibody-dependent enhancement (ADE), which can exacerbate disease symptoms^4^.

Virus fusion with host cell endosomal membranes, facilitated by a low pH, is a major event for certain virus infection cascade^6^. Vacuolar ATPase (V-ATPase) activity, which is responsible for pumping protons into endosomal compartments, has been identified as a requirement for feline coronavirus replication in previous studies^7^. The endosomal acidification process is also necessary for the establishment of ADE in FIPV infection^8^. Therefore, the endosomal V-ATPase presents an attractive target for antiviral intervention. In the past decades, abundant studies indicated that V-ATPase inhibitors could be applied to treat viral infections as well as other diseases including osteoporosis, lytic bone disease, renal acidosis, and cancer^9,10^. For example, the most commonly used V-ATPase inhibitor, bafilomycin, showed a broad-spectrum antiviral effect against several pathogens. However, the drug’s cytotoxicity compromises its clinical applicability^11^. More recently, diphyllin, a natural compound extracted from the plant *Cleistanthus collinus*, was discovered as a potent V-ATPase inhibitor with a promising safety profile. Studies have shown that diphyllin at nanomolar concentrations can inhibit V-ATPase activity in bovine chromaffine granules and lysosomal acidification in human osteoclasts with no cytotoxic effect on bone formation^12^. In a study on treating gastric adenocarcinoma, diphyllin decreased V-ATPase activity and lowered tumor cell infectivity for improved disease outcome^13^. A previous study also demonstrated diphyllin’s antiviral effect *in vitro* against influenza and dengue viruses^14^, highlighting the compound’s potential as a broad-spectrum “host-targeted” antiviral. The present study thus aims to investigate the compound’s effect against FIPV.

As drug safety and delivery are critical factors that determine an antiviral’s translational potential, a nanoformulation of diphyllin is herein developed with the aim of improving diphyllin safety and efficacy using poly(ethylene glycol)-block-poly(lactide-co-glycolide) (PEG-PLGA)^15^. The block-copolymer is highly biocompatible and frequently employed for drug delivery applications, and the nanocarrier may benefit the diphyllin compound in two ways. Firstly, the hydrophobic cores of PEG-PLGA nanocarriers offer an ideal medium for carrying and delivering the hydrophobic diphyllin compound, obviating the need for organic solvents. Secondly, the intracellular uptake of nanoparticles via the characteristic endocytosis mechanism may enhance diphyllin efficacy by facilitating compound colocalization with endosomal V-ATPase, thereby reducing the drug’s off-target effect and enhancing its antiviral activity. To examine the benefits of the diphyllin nanoformulation, cellular cytotoxicity and antiviral activity between free diphyllin and diphyllin nanoparticles were compared. In addition, an *in vitro* model of FIPV infection was established to assess the viral inhibitory effect of diphyllin nanoparticles in the context of ADE. Finally, safety of the diphyllin nanoparticles were assessed following intravenous injections in mice. Blood chemistry analysis and body weight monitoring were performed to evaluate the drug’s safety *in vivo*.

## Results

### Diphyllin blocked endosomal acidification in fcwf-4 cells

**Figure 1:**
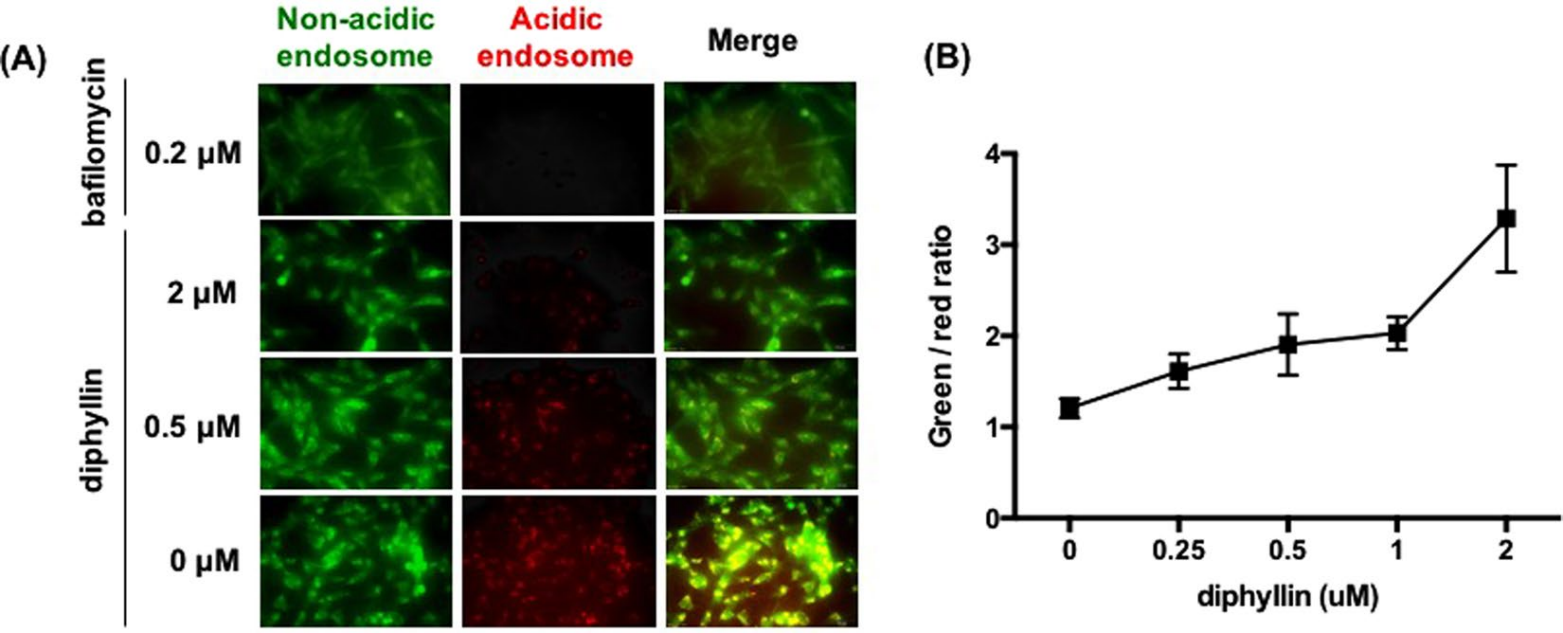
Dose-dependent inhibition of endosomal acidification caused by diphyllin. Fcwf-4 cells were incubated with bafilomycin A1 or various concentrations of diphyllin at 37 °C for 20 min. Untreated cells were used as controls. Acridine orange dye (1 μg/ml), which adopts a green fluorescence in non-acidic endosomes and a red fluorescence in acidic endosomes, was added to each well and incubated for 10 min. (**A**) Acidic endosomes in cells were stained red by acridine orange and non-acidic endosomes were stained green. Representative images are shown (magnification: 400×). (**B**) Fluorescence data was collected from diphyllin-treated wells and the green/red fluorescence ratio was presented. Data in the plot present the mean ± SEM out of four replicates.

Upon acridine orange labeling, intracellular acidic endosomes were stained red whereas non-acidic compartments appeared green under the fluorescence microscope. As shown in Fig. 1, in comparison to the extensively red-stained endosomes in untreated cells, the addition of bafilomycin A1 or diphyllin showed a decreased number of acidic endosomes and an increased number of non-acidic endosomes in cells. The degree of inhibition in endosomal acidification was shown to correlate with diphyllin concentration (Fig. 1A). To quantitatively analyze the endosomal acidification, green and red fluorescence data collected from diphyllin-treated wells were compared, and the green/red fluorescence ratio was indicated in Fig. 1B. A positive correlation was observed between the green/red ratio and diphyllin concentration, validating inhibition of endosomal acidification in fcwf-4 cells by the diphyllin compound.

### Pretreatment of diphyllin exhibits the maximal antiviral effect

**Figure 2:**
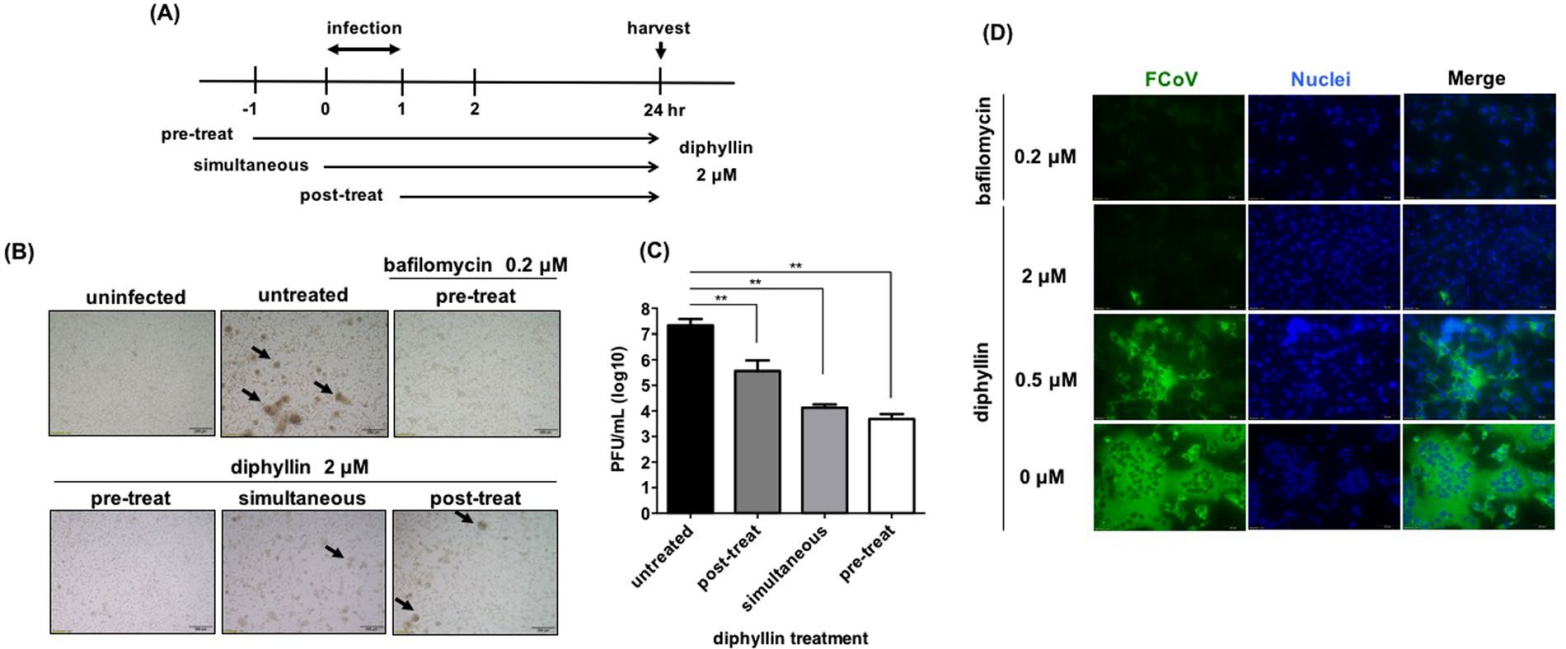
Diphyllin alters cellular susceptibility against FIPV. (**A**) Schematic presentation of the time-of-addition assays for diphyllin treatment. 2 μM of diphyllin was added to fcwf-4 cells at three different time points relative to FIPV virus infection (MOI = 0.0001): 1 h prior to infection (pre-treat), same time as infection (simultaneous) or 1 h after infection (post-treat). After a 1 h infection period, all test cells were washed and incubated with fresh media containing 2 μM of diphyllin and incubated for 24 h. (**B**) Cells were then harvested for CPE observation. Virus-induced CPE are indicated by arrows. Infected cells without diphyllin treatment (untreated) or 0.2 μM bafilomycin-treated cells were used as controls. (**C**) Extracellular viral titers in culture supernatant were determined with plaque assays. Viral titers between each treated group and the untreated control group were compared by one-way ANOVA followed by Dunnett’s multiple comparisons test (**p < 0.01). Data in the plot present the mean ± SEM out of three replicates. (**D**) 0.2 μM of bafilomycin A1 or various concentrations of diphyllin (0.25, 0.5, 1, 2 μM) were added to fcwf-4 cells one hr before FIPV infection (MOI = 0.0001). Infected cells without diphyllin treatment were used as controls. After a 1-hr period of infection, cells were washed, overlaid with fresh media containing the same concentrations of diphyllin as in previous step, and incubated for another 24 hr. Fluorescence images of FITC (green), which represents expression of viral nucleocapsid protein, and nucleus (DAPI, blue) were acquired. Representative images are shown (magnification: 400×).

2 μM of diphyllin was added to fcwf-4 cells at three different time points relative to virus infection (Fig. 2A). Compared to the untreated control, diphyllin treatment before, during, and after infection all resulted in cytopathic effect (CPE) inhibition (Fig. 2B) and reduced viral titers in culture supernatants (Fig. 2C). The inhibitory effect against the FIPV infection was most pronounced when the cells were exposed to diphyllin prior to viral exposure. Other incubation schedules also resulted in significant reduction in FIPV titer, thereby validating diphyllin’s antiviral ability. To further demonstrate the viral inhibition activity by diphyllin, immunofluorescence staining was performed. FITC-labeled antibodies were used to detect FIPV nucleocapsid protein under fluorescence microscopy. As seen in Fig. 2D, with the reference of the DAPI-stained nuclei, cytoplasmic fluorescent signal was reduced with pretreatment of bafilomycin A1 and varying concentrations of diphyllin. A dose-dependent inhibitory effect against FIPV was observed for the diphyllin compound.

### Characterization of diphyllin-loaded nanoparticles

**Figure 3:**
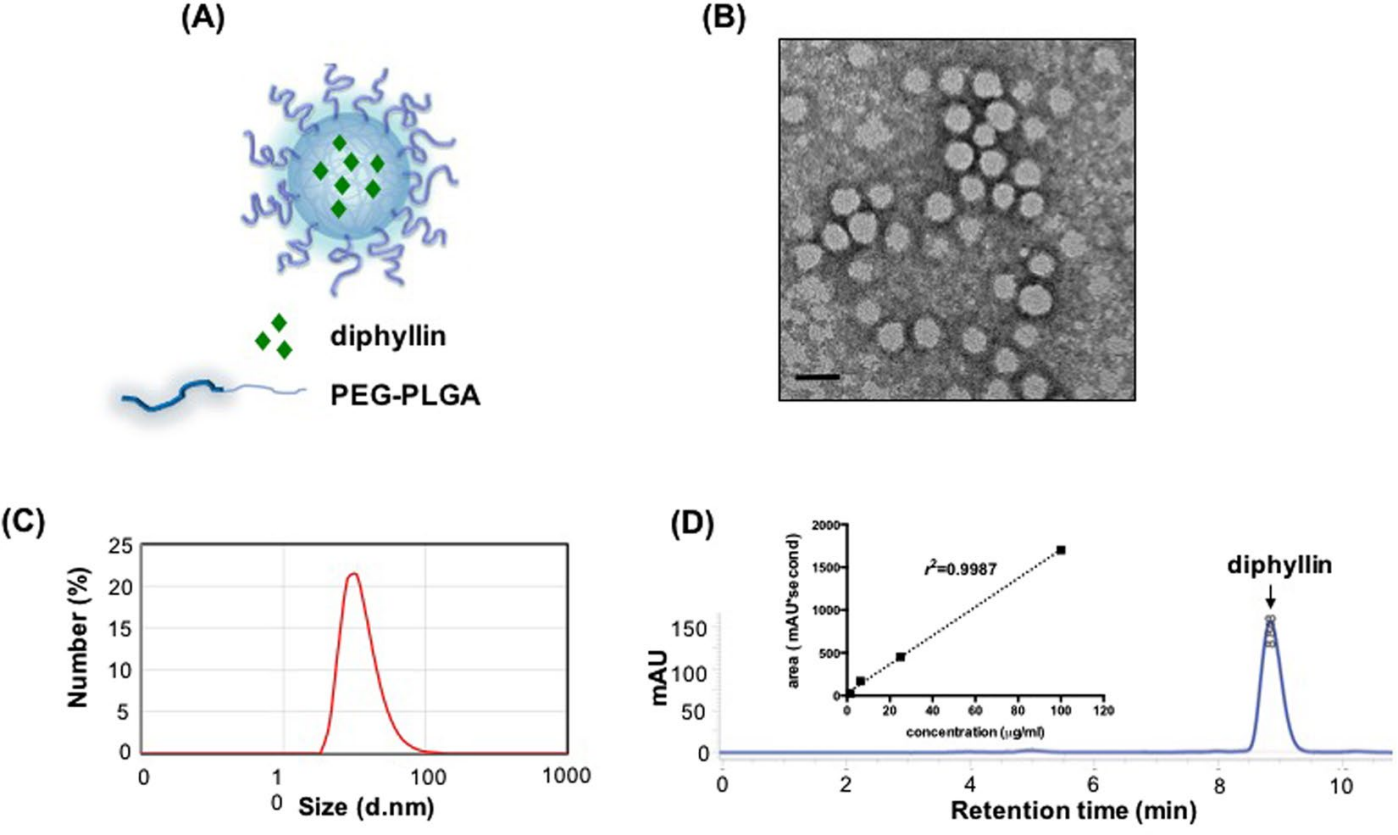
Preparation and characterization of diphyllin-encapsulated nanoparticles. (**A**) Illustration of diphyllin-encapsulated PEG-PLGA nanoparticles. (**B**) Diphyllin nanoparticles were visualized using transmission electronic microscopy under negative staining with uranyl acetate. Bars = 50 nm. (**C**) The nanoparticle size measurement by DLS. (**D**) The HPLC analysis of diphyllin (inset, standard curve of diphyllin).

The composition of diphyllin-loaded PEG-PLGA nanoparticles is illustrated in Fig. 3A. To examine the formation of nanoparticles, TEM analysis with negative staining was performed. Under the TEM visualization, diphyllin-load nanoparticles (Fig. 3B) were observed to be uniform in size with an average diameter of 40 nm. To further characterize the nanoparticles, DLS was used to analyze the size distributions of the nanoparticles. The results from DLS demonstrate that diphylin-loaded nanoparticle exhibited an average particle diameter of 36.19 ± 1.15 nm (Fig. 3C). Quantification of diphyllin loading in the PEG-PLGA nanoparticles was performed using HPLC to determine the drug loading yield and efficiency. As presented in Fig. 3D (arrowed peak), the elution of diphyllin was observed at 8.8 min in the chromatogram. The standard curve was constructed with serially diluted samples of diphylin (1.56, 6.25, 25 and 100 μg/mL) that separately corresponded to each area value 116.19537, 489.75905, 1997.97016 and 5392.25782 (mAU*second) (Fig. 3D inset). With the reference of standard curve, the content of diphyllin in nanoparticles for each batch was determined. In this study, the loading efficiency of diphyllin was 13.52% with a drug loading yield of 0.67 wt%.

### Safety and antiviral effect of diphyllin and diphyllin-loaded nanoparticles

**Figure 4:**
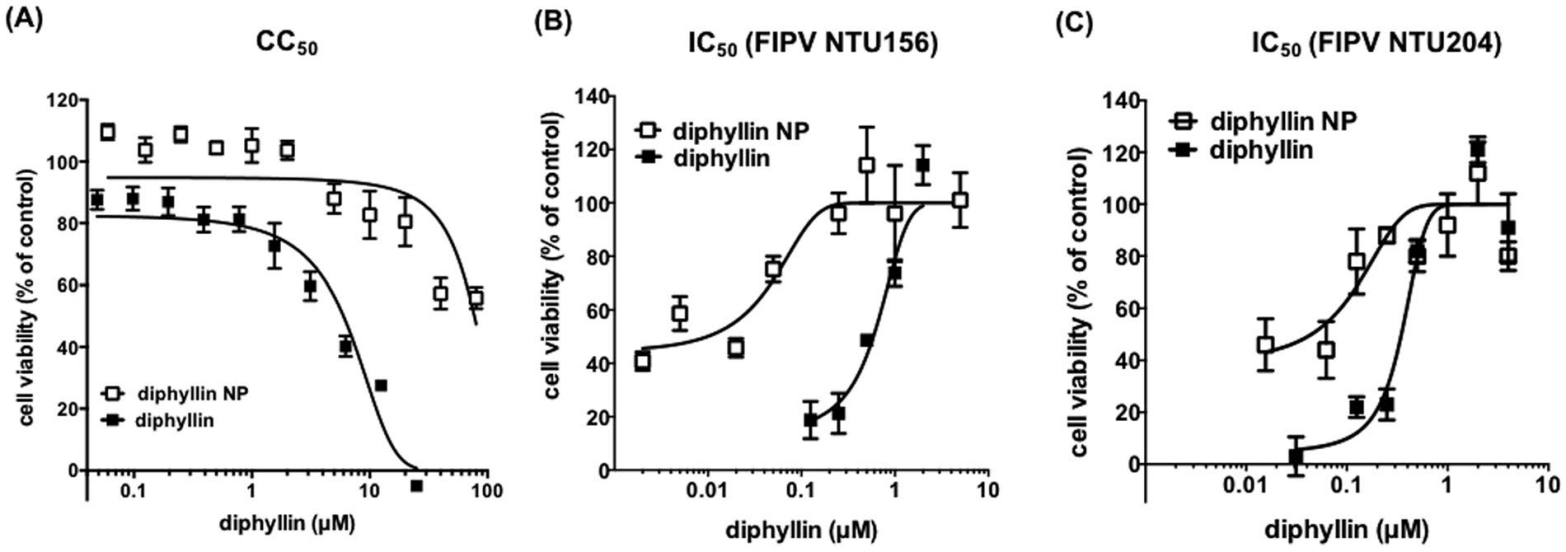
Dose-dependent cytotoxicity and anti-FIPV activity of diphyllin and diphyllin nanoparticles. (**A**) Various concentrations of diphyllin were added to fcwf-4 cells and incubated for 24 hr. An MTT assay was performed and cell viability was normalized to the value of untreated vehicle controls (100%). Data in the plot present the mean ± SEM out of four test replicates. (**B**) Various concentrations of diphyllin or diphyllin nanoparticles were added to FIPV (NTU156; MOI: 0.0001)-infected fcwf-4 cells and incubated for 24 hr. An MTT assay was performed and cell viability was normalized to the value of untreated vehicle controls (100%). Data in the plot present the mean ± SEM out of four test replicates. (**C**) Various concentrations of diphyllin or diphyllin nanoparticles were added to FIPV (NTU204; MOI: 0.01)-infected fcwf-4 cells and incubated for 24 hr. An MTT assay was performed and cell viability was normalized to the value of untreated vehicle controls (100%). Data in the plot present the mean ± SEM out of four test replicates.

Cell viability of fcwf-4 cells treated with various concentrations of diphyllin was measured using an MTT assay. After 24 hr of incubation, the CC_50_ of diphyllin was determined as 5.99 ± 0.68 μM in fcwf-4 cells, whereas the CC_50_ of diphyllin nanoparticles was 77.26 ± 13.14 μM (*p* < 0.0001) (Fig. 4A), indicating a 13-fold reduction in cellular toxicity with the nanoformulation. No reduction in cellular viability was observed with the empty nanoparticle control at concentrations up to 20 mg/mL (Supplementary Fig. S1), which is more than 4 times higher than the highest diphyllin nanoparticle formulation examined in the study (80 μM of diphyllin; 4.5 mg/mL of nanoparticles). The result demonstrates a significantly improved safety profile with the diphyllin nanoparticles as compared to the free diphyllin formulation.

To assess the IC_50_ of diphyllin, various non-toxic concentrations of diphyllin were used to treat FIPV infection using an FIPV NTU156 strain at an MOI of 0.0001. An MTT assay was performed to examine the CPE protection. As compared to the untreated control, the presence of diphyllin significantly inhibited the number of CPE foci induced by the viral infection. Based on the MTT assay result, the IC_50_ value of diphyllin against NTU156 at an MOI of 0.0001 in fcwf-4 cells was 590 ± 91 nM. The diphyllin nanoparticle, on the other hand, showed an IC_50_ of 9.751 ± 2.35 nM (*p* < 0.0001) (Fig. 4B), which corresponds to a 60-fold enhancement in antiviral activity as compared to the free drug. Empty PEG-PLGA nanoparticles showed no antiviral effect at 10 mg/mL (Supplementary Fig. S1). Calculation of therapeutic index (TI = CC_50_/IC_50_) yielded values of 10 and 7923 for the free diphyllin and diphyllin nanoparticles, respectively. The antiviral activities of diphyllin and diphyllin nanoparticles were further assessed against a different strain of FIPV (NTU204). At an MOI of 0.01, the free diphyllin and the diphyllin nanoparticles exhibited IC_50_ values at 348 ± 53.6 nM and 44.3 ± 38.3 nM respectively (*p* < 0.0001) (Fig. 4C). Enhancement of diphyllin antiviral efficacy by the nanoparticles was also observed at an MOI of 0.1 (1034 ± 49.5 nM vs 585 ± 61.9 nM in IC_50_ values; *p* < 0.001) (Supplementary Fig. S2), validating that the nanoformulations greatly broadened the therapeutic window of the compound.

### Antiviral activity of diphyllin-loaded nanoparticles in ADE model of FIPV infection

**Figure 5:**
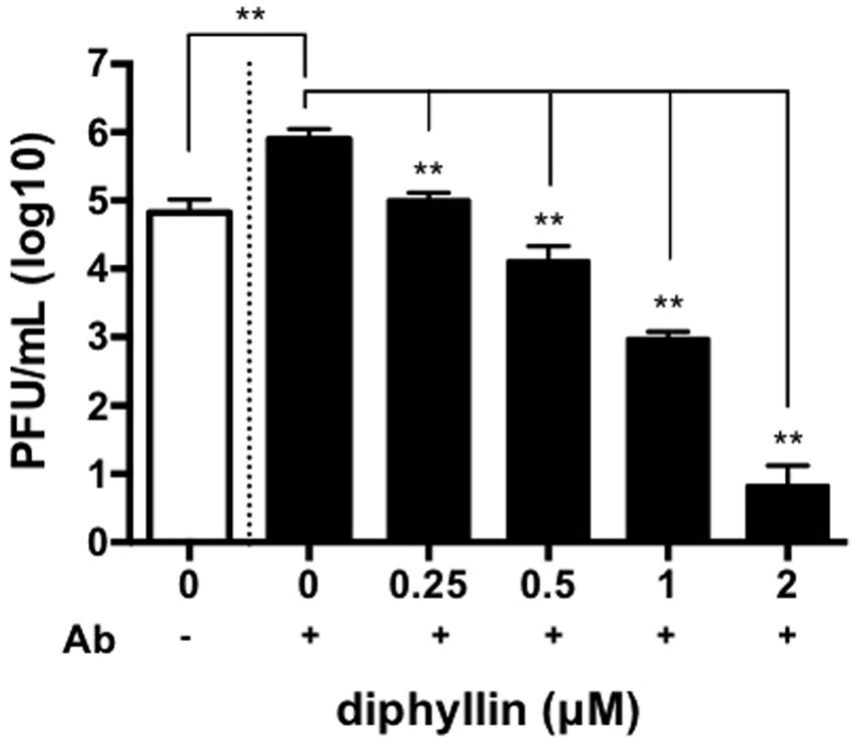
Various concentrations of diphyllin loaded PEG-PLGA nanoparticles were added to fcwf-4 cells in a direct infection (white open bar) or ADE infection (black bars) for 48 hr incubation (NTU156, MOI = 0.0035). The viral titers from the collected culture supernatants were titrated by plaques assays. Viral titers between each diphyllin-treated group and the untreated control group were compared by one-way ANOVA followed by Dunnett’s multiple comparisons test (**p < 0.01). Data in the plot present the mean ± SEM out of three replicates.

In light of the promising therapeutic index of the diphyllin nanoparticles, the nanoparticles’ antiviral activity was further examined against FIPV-infected cells under antibody-dependent enhancement. Plaque assays were conducted to examine the viral load in culture supernatants derived from two different infection conditions in the presence and absence of anti-FIPV mAb. As indicated in Fig. 5, the presence of mAb during viral infection resulted in a significant increase of viral titer from 6 × 10^4^ PFU/ml to 8 × 10^5^ PFU/ml in the culture supernatant. Fcwf-4 cells were infected with FIPV in the context of ADE, and the antiviral activity of diphyllin at various concentrations was tested by plaque assays after 48 hr of incubation. It was observed that diphyllin reduced antibody-enhanced FIPV infection in a dose-dependent manner, and 2 μM of diphyllin nanoparticle treatment reduced the viral load by 5 orders of magnitude to 6 PFU/ml. The result highlights the promise of diphyllin nanoparticles in addressing a serious immunopathological complication in FIPV infection.

### Diphyllin nanoparticles are well tolerated *in vivo*

**Figure 6:**
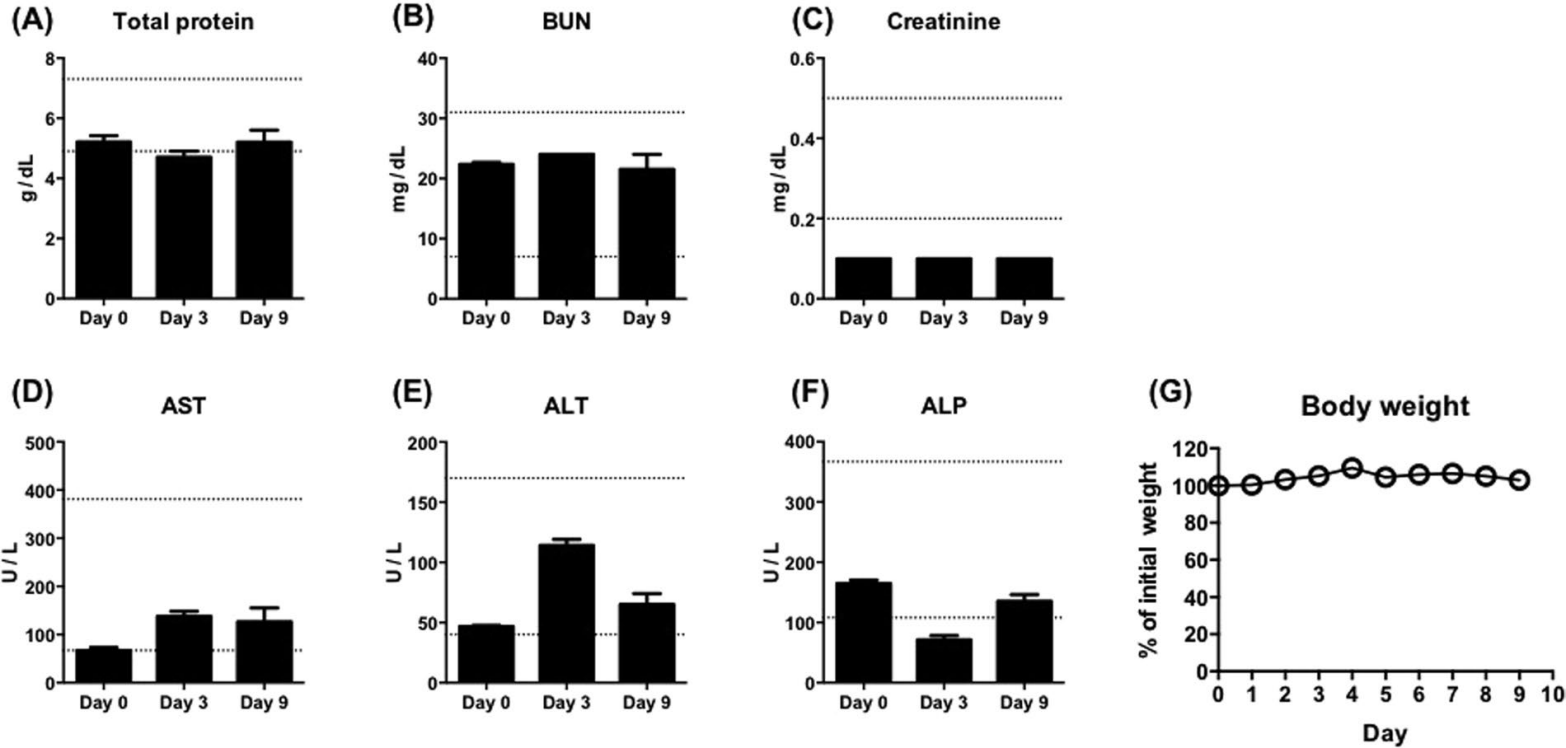
Mice intravenously injected twice with 100 μL of 80 mg/mL diphyllin nanoparticles (5 μg of diphyllin) were evaluated for changes in blood chemistry parameters, including (**A**) total protein, (**B**) blood urea nitrogen (BUN), (**C**) creatinine, (**D**) aspartate transaminase (AST), (**E**) alanine transaminase (ALT), and (**F**) alkaline phosphatase (ALP), and (**G**) body weight. Dashed lines represent normal ranges for BALB/c mice. Data in the plot present the mean ± SEM out of three mice.

To assess the safety of the diphyllin nanoparticles *in vivo*, blood chemistry analysis and body weight monitoring were performed on mice treated with the nanoformulation. 100 μL of 80 mg/mL diphyllin nanoparticle (5 μg of diphyllin) were intravenously given to 8-week-old BALB/c mice twice on day 0 and day 1, and blood draws were performed on day 0 before the nanoparticle administration, day 3, and day 9. Blood chemistry analysis showed that many parameters, including total protein, blood urea nitrogen (BUN), and creatinine were not affected by the nanoparticle administration (Fig. 6A–C). Aspartate transaminase (AST) and alanine transaminase (ALT) were observed to be elevated following the treatment but remained within normal ranges^16^ (Fig. 6D,E). The diphyllin nanoparticles were found to decrease the level of alkaline phosphatase (ALP) on day 3, but the effect was transient as the ALP level recovered by day 9 (Fig. 6F). Monitoring of body weight showed little weight change among the treated animals over 9 days (Fig. 6G). Overall, the diphyllin nanoparticles were well tolerated as the tested animals showed no sign of distress over the 9-day observation.

## Discussion

FCoV infection is common in young cats, and up to 12% of infected cats may develop FIP^17^. To this date, FIP remains fatal in most cases and lacks effective prophylactic and therapeutic treatment options. In clinical settings, recommended disease management during FIP manifestation includes a variety of supportive treatments, such as immunomodulatory drugs, antibiotics, anticoagulants, fluids and supplements^3^. Studies have shown that disease severity correlates with the systemic viral load^18^, hence, antiviral therapeutics that reduce the viral spread may be beneficial towards disease control. Previously, a coronaviral protease inhibitor GC376 demonstrated a promising advance in direct inhibition of virus replication in a clinical trial^19^. Other pathogen-targeted antiviral approaches have been tested *in vitro*, awaiting further efficacy validation *in vivo* ^20–25^. Among different antiviral strategies, host-targeted therapeutics are increasingly recognized as an attractive antiviral treatment approach as they do not exert direct selective pressures on viral pathogens. Advantages of host-targeted therapies include broad applicability against multiple viruses and insusceptibility to drug-resistant mutations^26,27^. Examples of host-targeted treatments against FIP include the use of cyclosporine A to suppress the replication of FIPV in cell culture via the inhibition of cyclophilin^28,29^. As cyclophilin is a critical host factor responsible for the replication of many members of the *Coronaviridae* family, cyclosporine A was suggested to be a pan-coronavirus inhibitor^30,31^. In another example, chloroquine was shown to have anti-FIPV and anti-inflammatory activities *in vitro* and further relieved clinical symptoms in FIP-infected cats. The compound, however, poses safety concerns and it may inflict liver damage^32^. Therefore, the present study explores a novel compound and an alternative delivery approach towards facilitating safe and effective anti-FIPV drug development.

Diphyllin, a new class of V-ATPase inhibitor, is herein applied for the inhibition of endosomal acidification for FIPV treatment. V-ATPase is a major class of cation translocating enzyme that is involved in a variety of vital processes, including endocytosis, protein trafficking, and metabolites transport. The compound has been previously applied as therapeutics, including treatments against cancer and leishmaniasis. It has also been demonstrated as a host-targeted treatment against infections by influenza and dengue viruses, reducing cellular entry by the viruses by intercepting the endosomal acidification process. The present study further validates the compound’s antiviral effect against FIPV, affirming the compound’s broad applicability as an antiviral agent. As V-ATPases are present diverse subunit isoforms in different organs and are involved in different disease pathogenesis, transient, tissue-specific inhibition of V-ATPases by the compound may open up new therapeutic opportunities.

In spite of reported complexity of virus internalization^33,34^, it has been recognized that acidity-mediated endosomal escape and cytosolic entry are essential pathways in the FCoV life cycle^7,8,35,36^. Medium to low sensitivity to the inhibitor of endosomal acidification (NH_4_Cl) exhibited by serotype II FCoV 79-1146 cultured in A-72 cells was reported by Regan *et al*.^7^, whereas our serotype II FCoV NTU156 and NTU204 cultured in fcwf-4 cells has shown significant reduction when treated with bafilomycin A1, consistent with Takano *et al*.^8^, or diphyllin. Differential role for acidic endocytic compartments in cellular entry between these two FIPV strains suggests that the low-pH entry may be strain-dependent and biotype-independent. While diphyllin also exhibited inhibitory effect on NTU156 cultured in U937 cells under the ADE infection (Supplementary Fig. S3), we speculate the sensitivity to dipyllin exhibited by NTU156 is cell type-independent. In addition, type II FCoV 79-1146 cultured in fcwf-4 cells was found less sensitive to the treatments of viroporin inhibitors and cholesterol^24,37^.

Towards applying diphyllin for therapeutic purposes, the compound’s insolubility and potential adverse effect are two concerns that can be addressed using nanocarrier technology. Nanoparticle-based drug delivery presents unique advantages that arise from the carriers’ nanoscale morphology and drug loading capacity. The approach has been applied extensively in anticancer applications, yielding formulations with improved delivery efficiency and therapeutic efficacy^15,38–40^. More recently, nanomedicine platforms have begun to draw attention as an antiviral delivery platform. For example, acyclovir, a common antiviral drug against herpes simplex virus type-1 (HSV-1), was delivered by nanocarriers for enhanced efficacy and reduced side effects, exhibiting a three-fold improvement in therapeutic effect in comparison to free drugs^41^. Saliphenylhalamide (Saliphe), a V-ATPase inhibitor, has also been shown to display enhanced viral inhibition when delivered via nanoparticles^42^. The virus-like dimension of nanoparticles may favor drug delivery to sites of viral infections as many similarities regarding cellular entry and *in vivo* biodistributions have been observed between viruses and nanocarriers^43^. In the present study, PEG-PLGA, a biocompatible block co-polymer widely used for drug delivery applications^44–47^, was applied for the encapsulation and delivery of diphyllin. The nanoparticles significantly improved the safety and efficacy of the diphyllin compound, increasing the therapeutic index by approximately 800-fold in one of our infection models. This pronounced enhancement can be attributed to multiple characteristics of the nanomaterial. Firstly, the hydrophobic nature of the PEG-PLGA nanoparticle cores facilitates diphyllin incorporation and obviates the need of organic solvents for compound dissolution. As a result, rather than permeating through the cells with the aid of DMSO, nanocarrier-encapsulated diphyllin relies on nanoparticle-mediated endocytosis for cellular entry. V-ATPase, the target of diphyllin, are ubiquitous among intracellular compartments and govern a multitude of physiological cellular functions^10^. Enhancing diphyllin localization inside endosomes may thus reduce the compound’s potential side effects. Higher drug efficacy and improved cell protection by the nanoformulations can also be directly attributed to enhanced endosomal drug delivery by the nanoparticles. A higher effective dosage can efficiently reach the endosomal protein target upon nanoparticle delivery.

**Figure 7:**
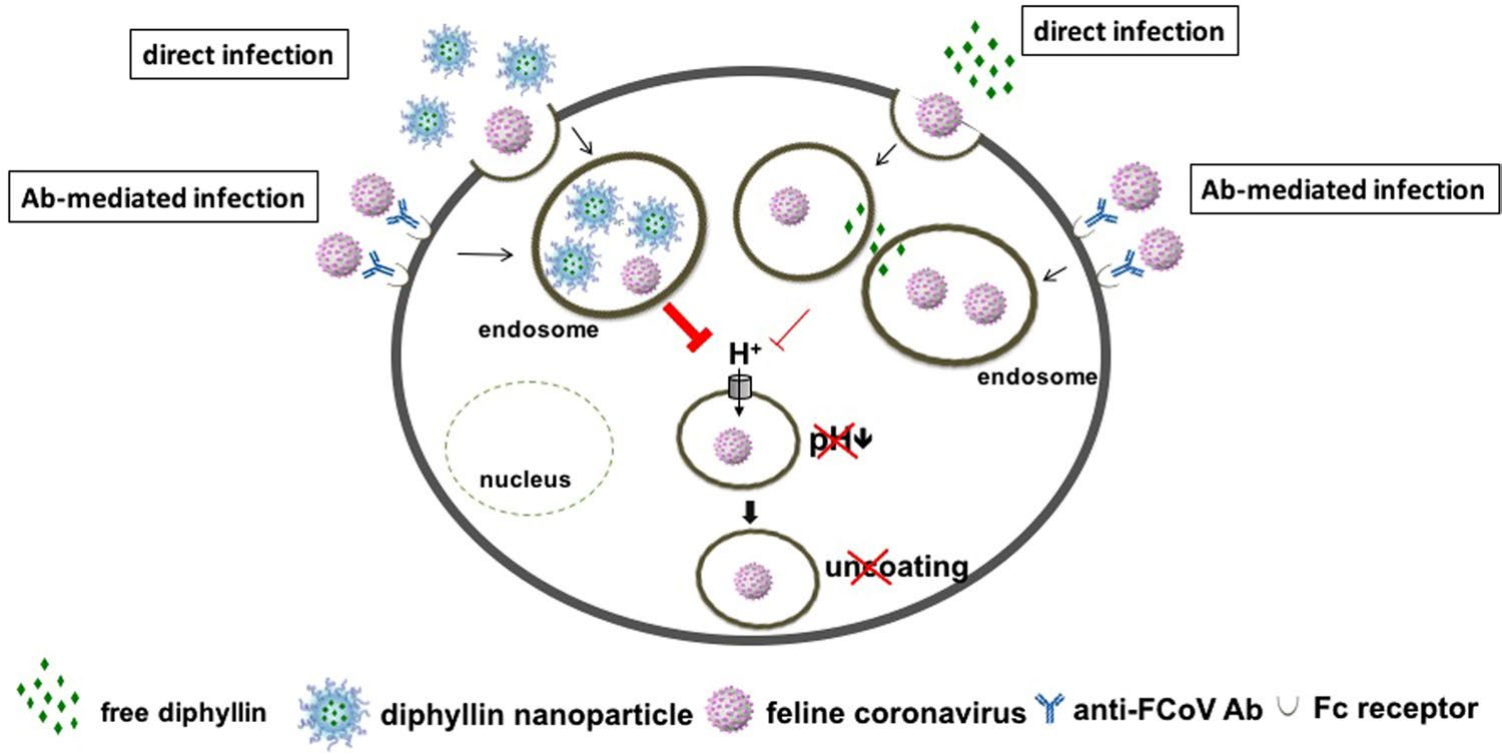
Schematic diagram of diphyllin’s antiviral mechanism. Diphyllin demonstrated prominent inhibitory activity in anti-FIPV infection by attenuating cellular organelle acidification, thus blocking virus entry. In addition, diphyllin treatment displayed suppression in viral load in the context of ADE of FIPV infection. By using a nanoparticulate drug delivery system, diphyllin exhibited improved safety and enhanced antiviral activity.

As an immune-related disease, FIPV possesses an ADE characteristic that contributes to immunopathology and complicates disease prognosis. The recognition of FIPV immune-complexes via the FcR by immune cells can enhance disease progression through altered cross-reactive cellular immune response, inefficient lysis of infected cells, and altered T cell-cytokine responses that collectively promote ADE. Although the relationship between serotypes or strains and the ADE phenomenon remains unclear^48^, the requirement of endosomal acidification for ADE has been well established^8^. Our study has demonstrated that diphyllin nanoparticles possess dose-dependent antiviral activity in an *in vitro* model of ADE of FIPV infection, which mimics the disease’s clinical manifestation. By attenuating cellular organelle acidification, the nanoformulation effectively inhibits endosomal acidification responsible for virus uncoating and cytoplasmic entry (Fig. 7). As the diphyllin nanoparticles are shown to be well tolerated *in vivo* upon high-dose intravenous administration, the formulation presents a promising treatment option against the lethal feline disease. Future studies with *in vivo* animal models of viral infections are warranted.

## Conclusions

Diphyllin, a V-ATPase inhibitor, is herein demonstrated to reduce FIPV infection by inhibiting the endosomal acidification process that is required for virus uncoating and cellular entry. Diphyllin pretreatment to cells prior to viral infection altered the cellular susceptibility to the virus and maximized the compound’s antiviral activity. To enhance the translational potential of the antiviral compound, PEG-PLGA nanoparticles were applied for drug encapsulation and delivery. The nanoformulation significantly reduced the toxicity and increased the antiviral effect of diphyllin, enhancing the compound’s therapeutic index by up to 800-fold in *in vitro* analysis. The diphyllin nanoparticles potently inhibits FIPV infection in an *in vitro* ADE model. In an animal study, the diphyllin nanoparticles were well tolerated following intravenous administration. The present work highlights diphyllin and diphyllin nanoparticles as effective host-targeted antivirals. It presents a promising treatment modality against lethal infectious diseases.

## Methods

### Cells

Fcwf-4 (felis catus whole fetus-4) cell line was purchased from ATCC and maintained at 37 °C with 5% CO_2_ in Dulbecco’s modified Eagle’s medium supplemented with 10% fetal bovine serum and 1% penicillin/streptomycin/amphotericin B. U937 cell line was purchased from the Bioresource Collection and Research Center (Hsinchu, Taiwan) and maintained at 37 °C with 5% CO_2_ in RPMI-1640 medium supplemented with 10% fetal bovine serum and 1% penicillin/streptomycin/amphotericin B. All the culture reagents were purchased from Gibco.

### Viruses, plaque assays, and quantitative RT-PCR

The serotype II FIPV strains NTU156^49^ and NTU204 isolated at National Taiwan University were used in this study. The viruses were propagated and titrated in fcwf-4 cells. The titrated viral stocks were stored in −80 °C. For plaque assays, fcwf-4 cells were seeded in 12-well plates at 2 × 10^5^ cells/well one day prior to experiment. Right before experiment, media were removed and cells were washed. Virus-containing samples were ten-fold serially diluted with media, and 500 μl of the diluted samples were added to cells. After incubation at 37 °C for 1 hr, media were again removed and cells were washed. 1 mL of DMEM containing 1.8% methylcellulose (Sigma) and 2% FBS was added to each well. After 3 days, the plates were fixed with 10% formalin and stained with 1% crystal violet at room temperature for 10 min. Upon drying, the viral plaques were counted, and the plaque-forming unit (PFU) per ml of virus was determined. For FIPV infection in U937 cells, quantitative RT-PCR (qRT-PCR) was used for virus quantification. Briefly, viral RNA was extracted using a viral nucleic acid extraction kit (Geneaid Biotech Ltd., Taipei, Taiwan). cDNA was synthesized using a previously described P1b primer, and qPCR was performed using the primer set P009 and P010, and the fluorescence probe P9/10^50^. The cycling protocol in a thermal cycler (Biorad CFX Connect) consisted of 3 min of preheating at 95 °C, and followed by 35 cycles of 15 s of denaturation at 95 °C, 1 min of primer annealing and extension at 60 °C. 2 μL of plasmid DNA containing various copy numbers of membrane-nucleocapsid protein gene of FIPV was used for standard curve construction in qRT-PCR for viral load quantification. Two replicates were performed for each samples or standards.

### Diphyllin and diphyllin-encapsulated nanoparticles

Diphyllin (ChemDiv, San Diego, CA) and bafilomycin A1 (Sigma) were dissolved in dimethyl sulfoxide (DMSO, Sigma). For nanoparticle preparation, diphyllin was dissolved in chloroform at 1 mg/ml with 20 mg/mL of PEG-PLGA (Sigma). 1 mL of the solvent mixture was added to 5 mL of deionized water and probe sonicated for 1 min and 45 sec. The resulting emulsion was then placed in a fume hood for chloroform evaporation for 3 hr. For the vehicle control, empty PEG-PLGA nanoparticle was prepared in the absence of diphyllin. Particles were washed for 3 times using the Amicon Ultra 15 mL Centrifugal Filters (100k MWCO; Millipore) at 2000 × g for 4 min. Nanoparticle size distribution was measured by DLS (Malvern Instruments Inc., UK) based on the manufacturer’s instructions. The washed particles were then resuspended in 10% sucrose and stored at −80 °C.

### Analysis of endosomal acidification with acridine orange labeling

Fcwf-4 cells (4 × 10^4^ cells/well) were cultured onto an 8-well chamber slide (Nunc) one day before the experiment. Each well was incubated with various concentrations of diphyllin or 0.2 μM of bafilomycin A1 at 37 °C for 20 min. Bafilomycin A1 was used as a positive control, and 2% DMSO was applied as vehicle controls. Acridine orange dye (1 μg/ml; Biotium) was added to each well and incubated at 37 °C for 10 min before wash. Fluorescence images were obtained by fluorescence microscopy, and the red and green fluorescence colors were acquired using Texas Red and FITC filters respectively. Fluorescence intensity (green/red ratio) in each well was analyzed by ImageJ.

### Time-of-addition assay

Fcwf-4 cells were seeded in a 12-well plate at a density of 2 × 10^5^ cells/well 1 day before the experiment. 2 μM of diphyllin was added to the cells at three different time points with respect to virus infection. The time points include 1 hr prior to infection, same time as infection, and 1 hr after infection. Cells treated with 2% DMSO were prepared as controls. Virus (NTU156) was inoculated at an MOI of 0.0001. After 1hr of virus incubation, all cells were washed and resuspended in fresh media containing 2 μM of diphyllin. After 24 h, the cellular supernatants were collected, and virus titers in the supernatants were assessed by plaque assays.

### Immunofluorescence staining of intracellular viral protein

The immunofluorescence staining was performed to validate the antiviral effect of diphyllin. Fcwf-4 cells were seeded onto an 8-well chamber slide (4 × 10^4^ cells/well) one day before the experiment. After being treated with various concentrations of diphyllin for 1 hr, the cells were infected with FIPV-NTU156 at an MOI of 0.0001. Each well was fixed by 80% acetone at −20 °C for 20 min and air-dried at room temperature. Mouse anti-FCoV monoclonal antibody (clone: FIPV3-70; Biorad) was used as the primary antibody stain. FITC-conjugated goat anti-mouse IgG (Jackson Lab) was used as the secondary antibody stain. DAPI-containing mounting media (Vector shield) was then added to the antibody-stained cells, and the cells were observed using a fluorescence microscope.

### Transmission electronic microscopy

For transmission electron microscopy, 10 μL of nanoparticle sample was added onto a glow-discharged grid. The grid was then washed with ddH_2_O. Subsequently, the grid was stained with 1% uranyl acetate for 15 sec and excessive staining was removed by filter paper. The grid was air-dried overnight and stored in the desiccator until TEM observation using an FEI 120 kV Sphera microscope (FEI Tecnai F20).

### Quantification of diphyllin by HPLC

The content of diphyllin in the PEG-PLGA nanoparticles was quantified by HPLC (Agilent 1100 series). 200 μl of control PEG-PLGA nanoparticles and particles loaded with diphyllin were dissolved with 800 μl of acetone, which was then air-dried with nitrogen. The samples were then dissolved in mobile phase solvent and filtered prior to HPLC. Ascentis C18 column was equipped while mobile phase was prepared using methanol and water in proportion of 60% and 40%. The optimal detection wavelength was 254 nm and column temperature was 25 °C. Serially diluted samples of diphylin (1.56, 6.25, 25, 100 μg/mL) dissolved in the mobile phase solvent were prepared as standards for the HPLC analysis. The encapsulation efficiency (EE) of diphyllin was calculated using the formula: EE = (encapsulated drug/drug input) × 100%. The drug loading yield was calculated using the formula: loading yield = (weight of encapsulated drug/total polymer weight) × 100%.

### *In vitro* cytotoxicity of free diphyllin and diphyllin nanoparticles

The *in vitro* cytotoxicity of diphyllin and diphyllin nanoparticles was measured by an MTT assay. To obtain 80% confluent cell monolayers, fcwf-4 cells (2 × 10^4^/well) were seeded in 96-well plates with 100 μl of medium one day before the experiment. Cells were treated with different concentrations of diphyllin dissolved in 2% DMSO or diphyllin nanoparticles. After 24 hr, the culture supernatants were removed. 20 μl of 5 mg/ml MTT solution (3-(4,5-dimethylthiazol-2-yl)-2,5-diphenyltetrazolium bromide) was added to each well and the cells were cultured at 37 °C for 4 hr. The supernatant was removed and 100 μl of DMSO was added to each well to solubilize the purple formazan crystals. After 20 min, the absorbance was measured at 540 nm and 650 nm using a microplate reader. Untreated cells containing 2% DMSO were prepared to represent 100% cell viability. When testing the diphyllin nanoparticles, empty PEG-PLGA nanoparticles were used as the vehicle control. The concentration of diphyllin that reduced cell viability by 50% (CC_50_) was analyzed using GraphPad Prism (GraphPad Software, San Diego, CA).

### Cell cytopathic effect (CPE) inhibition assay and determination of IC_50_

To examine the antiviral effect of diphyllin, IC_50_ of diphyllin (the concentration of diphyllin showing 50% inhibition of virus-induced CPE) against FIPV-NTU156 was assessed using the cell viability assay. Briefly, fcwf-4 cells were seeded on 96-well microplates (2 × 10^4^ cells/well) one day prior to experiment to obtain an 80% confluent cell monolayer. Diphyllin was twofold diluted and added to fcwf-4 cells for 1 hr before virus inoculation. After 1 hr period of infection with a multiplicity of infection (MOI) of 0.0001 with FIPV NTU156, each well was washed and overlaid with media containing diphyllin of the same concentrations as in the initial treatment step. An alternative FIPV-NTU204 strain was tested using the same infection protocol with an MOI of 0.01 and 0.1. After another 24 hr of incubation, cellular viability was examined by an MTT assay. Signals from cells without infection and diphyllin treatment were used to represent 100% cell viability. The concentration of free diphyllin and diphyllin nanoparticles that inhibited virus-induced CPE by 50% (CC_50_) was determined by fitting the data onto a non-linear regression curve using GraphPad Prism (GraphPad Software).

### Establishment of *in vitro* antibody-dependent enhancement (ADE) infection of FIPV and the antiviral activity of diphyllin under the ADE infection

Upon addition of the mAb 6-4-2, the FIPV infection can be enhanced via Fc receptor (FcR)^8^. To set up the ADE infection condition, 1 mL of NTU156 viral suspension (1.4 × 10^4^ PFU) was mixed with 1 mL of the mAb 6-4-2 (1:10 diluted hybridoma supernatant) in a 15-mL centrifuge tube at 4 °C for 1 hr on a rotator. Subsequently, 0.1 mL of the resulting mixture was inoculated to fcwf-4 or U937 cells (2 × 10^5^ cells/well in a 12-well plate), which were pretreated with diphyllin for 1 hr. Bafilomycin A1 was used as a positive control. After the virus-antibody complex was added to cells and co-cultured for 3 hr (MOI = 0.0035), the cells were washed 3 times and re-incubated in diphyllin-containing fresh media. 48 hr later, supernatant from each well was collected and viral titers were determined by a plaque assay or qRT-PCR.

### Diphyllin nanoparticle safety in mice

8-week-old female BALB/c mice were intravenously administered with 100 μL of 80 mg/mL diphyllin nanoparticle (5 μg of diphyllin) twice on day 0 and day 1. Blood were withdrawn from the mice on day 0 before the nanoparticle administration, day 3, and day 9 for blood chemistry analysis. The mice were monitored for signs of distress and change in body weight for 9 days. The animal protocol was reviewed and approved by the Institutional Animal Care and Use Committee (IACUC) of the National Taiwan University (approval no. NTU105-EL-00181). All animal experiments were carried out in accordance with the approved guidelines.

### Statistical analyses

Data were analyzed by unpaired t tests or ANOVA by Dunnett’s multiple comparison tests using GraphPad Prism (GraphPad Software). The *p* values smaller than 0.05 were considered significant.

### Data availability

The datasets generated during the current study are available from the corresponding author on reasonable request.

## Electronic supplementary material

Supplementary information
