# Supplementary material for: Nanoparticulate vacuolar ATPase blocker exhibits potent host-targeted antiviral activity against feline coronavirus
Source: Sci Rep. 2017 Oct 12;7:13043. doi: 10.1038/s41598-017-13316-0 (PMC5638965; doi:10.1038/s41598-017-13316-0)

Nanoparticulate vacuolar ATPase blocker exhibits potent host-targeted antiviral activity against feline coronavirus

Che-Ming Jack Hu[1,2#], Wei-Shan Chang[3#], Zih-Syun Fang[1,3], You-Ting Chen[3], Wen-Lin Wang[3], Hsiao-Han Tsai[1,3], Ling-Ling Chueh[3], Tomomi Takano[4], Tsutomu Hohdatsu[4], Hui-Wen Chen[2,3]*

[1]Institute of Biomedical Sciences, Academia Sinica, Taipei, Taiwan
[2]Research Center for Nanotechnology and Infectious Diseases, Taipei, Taiwan
[3]Department of Veterinary Medicine, National Taiwan University, Taipei, Taiwan
[4]School of Veterinary Medicine, Kitasato University, Towada, Aomori, Japan

Supplementary Figure S1. (A) Various concentrations of empty PEG-PLGA nanoparticles were added to fcwf-4 cells and incubated for 24 hr. An MTT assay was performed and cell viability was normalized to the value of untreated cells (100%). Data in the plot present the mean ± SEM out of four test replicates. (B) Empty PEG-PLGA nanoparticle showed no antiviral activity against FIPV in fcwf-4 cells. Data in the plot present the mean ± SEM out of three test replicates. An unpaired t-test was used for the statistical analysis.

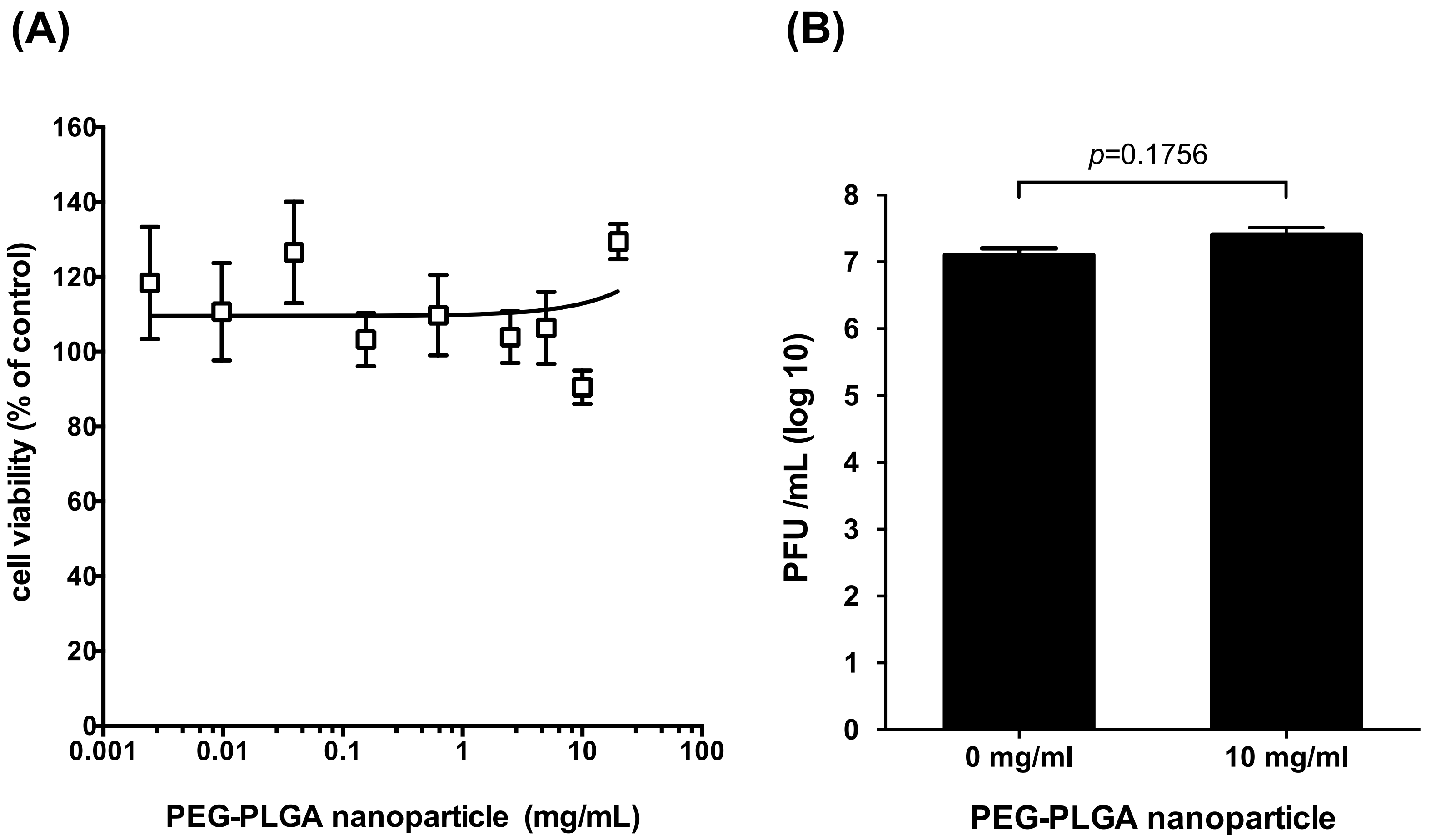

Supplementary Figure S2. Various concentrations of diphyllin or diphyllin nanoparticles were added to FIPV (NTU204; MOI: 0.1)-infected fcwf-4 cells and incubated for 24 hr. An MTT assay was performed and cell viability was normalized to the value of untreated vehicle controls (100%). Data in the plot present the mean ± SEM out of four test replicates.

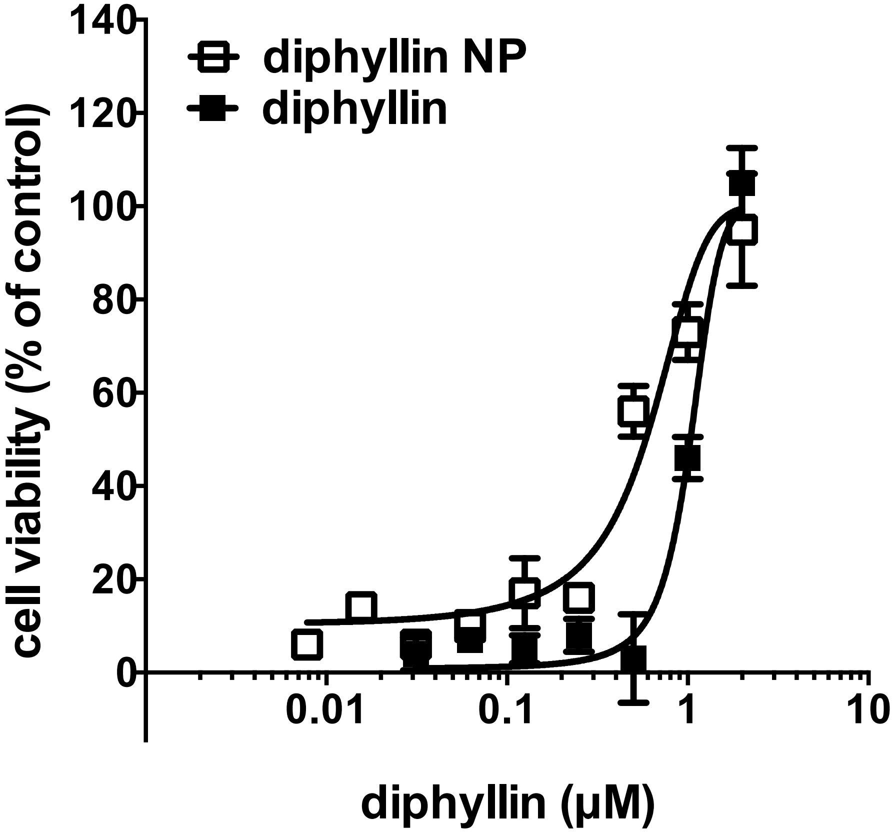

Supplementary Figure S3. Various concentrations of diphyllin were added to U937 cells in a direct infection (white open bar) or ADE infection (black bars) for 48 hr incubation (MOI = 0.0035). The viral titers from the collected culture supernatants were titrated by quantitative RT-PCR. Viral titers between each diphyllin-treated group and the untreated control group were compared by one-way ANOVA followed by Dunnett's multiple comparisons test (ns: non-significant, **$p < 0.01$, ***$p < 0.001$). Data in the plot present the mean ± SEM out of three replicates.

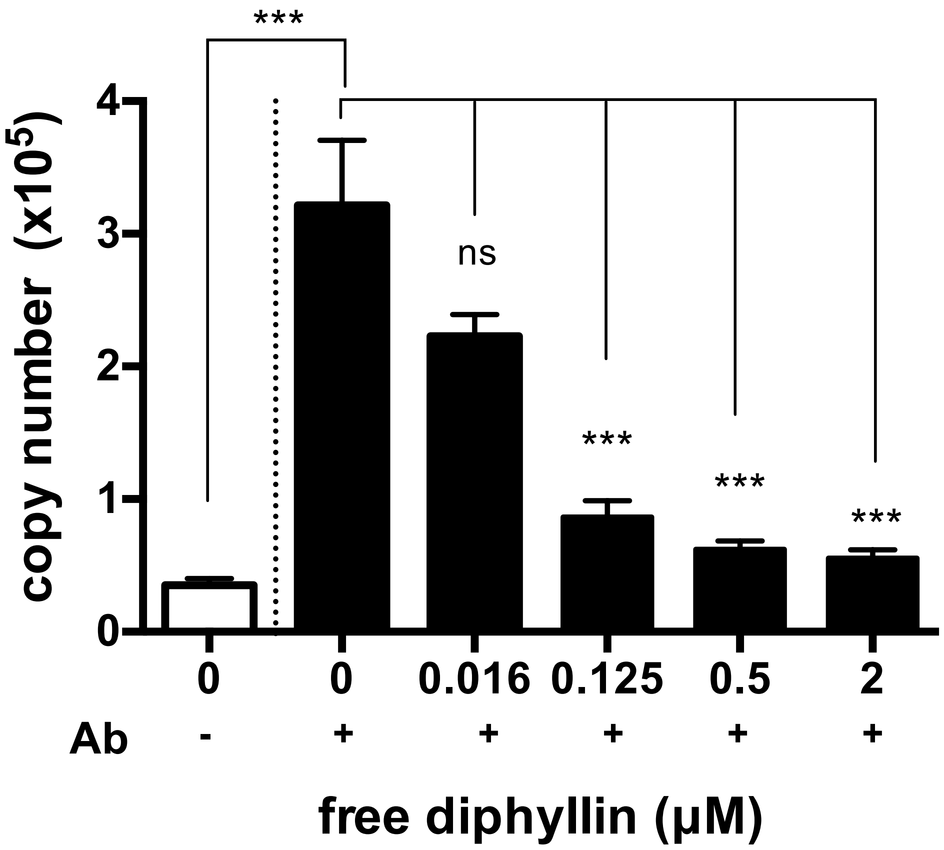

Supplement: Supplementary file 1 — Supplementary information [file 41598_2017_13316_MOESM1_ESM.pdf]
